# Plasma disinfection procedures for surfaces in emergency service vehicles: a field trial at the German Red Cross

**DOI:** 10.1038/s41598-023-47759-5

**Published:** 2023-11-25

**Authors:** Tom Schaal, Ulrich Schmelz

**Affiliations:** 1 University of Applied Sciences Zwickau, Zwickau, Germany; 2 University of Fulda, Fulda, Germany

**Keywords:** Bacteriology, Fungi, Health occupations

## Abstract

The demand for thorough disinfection within ambulances is essential, given the in-vehicle medical procedures and the potential high risk of infections due to patients' open wounds. One solution that can address this hygiene challenge involves the application of reactive products generated from atmospheric (air) oxygen and water vapor, activated through the use of cold plasma. Cold plasma's charged particles perforate the cell membranes of microorganisms. This process does not work in human cells, as proteins in the form of enzymes within the body break down the cold plasma and protect the cells. The study was done on an ambulance that was contaminated in eight places. Samples were taken from each site, and two surfaces measuring approximately 8 × 8 cm were carefully sealed and marked. These surfaces were deliberately contaminated by applying an Enterococcus faecium suspension of 8.5 × 107 CFU/mL using a sterile cotton swab. It was followed by the disinfection procedure, that was initiated with the PLASMOCAR device. It was positioned on the front workspace and operated for a duration of 30 min, utilizing the vehicle's onboard voltage. Throughout the operation, all doors and windows were closed and the vehicle's air conditioning system remained active. After the completion of the disinfection process, samples were collected from the surfaces for bacterial counts. A reduction of 3.73 log levels in initial bacteria was accomplished within the rescue vehicle for Enterococcus faecium, equivalent to a 10–fourfold reduction in bacteria, eliminating up to 99.99% of the initial microorganisms. This success makes the process well-suited and convenient as an ongoing "background" procedure to enhance the established disinfection procedures. The established disinfection procedures outlined in the hygiene plan must be promptly implemented whenever mechanical surface cleaning is required. The use of PLASMOCAR offers an extra layer of protection and security, significantly decreasing the risk of microorganism transmission through cross-contamination and aerosols. This is a significant benefit for the well-being of both staff and patients.

## Introduction

In emergency medical services, hygiene measures take place within well-planned routine transports and the urgent, time-sensitive demands of acute emergencies under a significant time pressure and in a rapidly changing environment. Due to the unpredictability of transmissible diseases or pathogens in emergency situations, as well as the unavailability of reliable preclinical information regarding the presence of such threats, the consistent compliance of fundamental hygiene measures becomes a requirement across all operations. This means that hygiene measures in emergency services must be fundamentally effective, appropriate, suitable for the situation, feasible, and cost-efficient. For this reason, hygiene standards must be established to minimize the risk of nosocomial infections of the patient as well as the transmission of pathogens to the emergency personnel^1,2^. The popularity of fogging devices for disinfection surged during the COVID-19 pandemic. However, their applicability in routine rescue services appeared to be unacceptable due to the fogging time of 30 to 90 min, Similarly, it could be stated that fundamental prerequisites for handling highly infectious patients are not material resources alone, but in particular the safe mastery of the required procedures^1,3^. Karl et al.^4^ were able to show a discrepancy between the 10 most frequently touched surfaces in the ambulance and the 10 most frequently disinfected surfaces. As a result, following Vikke et al.^5^, the potential danger of smear disinfection for ambulance personnel and subsequently patients to be transported as well as the possibility of germ reduction in periods without disinfection, e.g., by antimicrobial coatings, were discussed^4^.

PLASMOCAR uses the physical cold plasma technology^6–9^. Only ambient air and electricity are required to produce the cold plasma. Specifically, oxidative reaction products are generated from atmospheric oxygen and water vapor, which eliminate microorganisms and preserve human cells^10–12^. The cell membrane of the microorganisms is perforated by the charged particles of the cold plasma^13^.

The use of the PLASMOCAR can be described as harmless from three different perspectives:

### Medical safety

**Figure 1:**
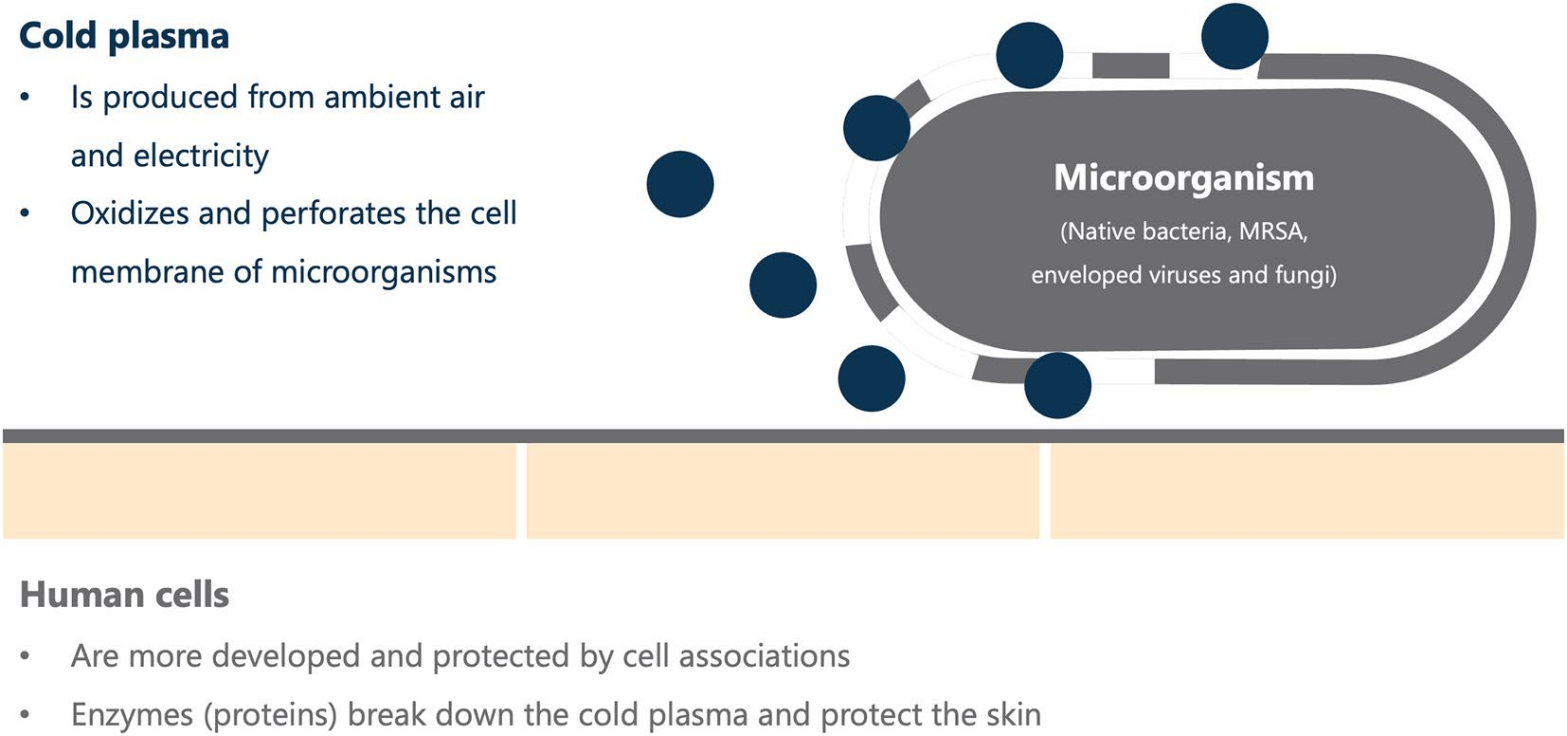
Eliminates microorganisms and preserves human cells.

PLASMOCAR can eliminate microorganisms and preserve human cells (Fig. 1). Reason for this is the physical process, which requires only ambient air and electricity to produce the cold plasma. The cell membrane of the microorganisms is perforated by the charged particles of the cold plasma. This is impossible in human cells because proteins in the form of enzymes break down the cold plasma and protect the cells^14,15^.

### Toxicological safety

The medical justification can be supported by toxicity tests. For instance, an Ames (EN ISO 10993-3) and a Cytotox test (EN ISO 10993-5) were performed for the cold plasma process on which PLASMOCAR is based. In both tests, no mutagenic effect was found in human cells.

### Legal safety

PLASMOCAR is based on a purely physical process^8,16^. Against this background, the Low Voltage Directive for electrical equipment applies. The Low Voltage Directive specifies emission limits such as the emission of the substance ozone (IEC 60335-2-65). This directive is supported by a recommendation from the VDI (Association of German Engineers). The expert recommendation outlined in VDI-EE 4300 Sheet 14 prescribes that air purification devices should not exceed an emission of a maximum residual ozone concentration of 10 μg/m3. The assessments conducted by CeCert company, dated 21.12.2021, as well as the assessment done by TÜV NORD Umweltschutz GmbH & Co. KG, dated 22.06.2022, provide confirmation of compliance with these prescribed limit values. "Disinfection" describes a microbiocidal process whose aim is to reduce an initial microbial load to such an extent that there is no longer any risk of infection from the remaining, residual microorganisms. This distinguishes disinfection from sterilization, whose goal is "absolute sterility" (with a statistical certainty of 1:1,000,000 for a non-sterile product – > SAL = sterility assurance level). The germ reduction performance is expressed in log levels, which as an exponent to the base 10 describe by how many times the germ reduction has occurred^17,18^.

Log reduction factors of 3 to 5 are recommended for disinfection processes, whereby a log reduction factor of > 3 should be present for surface disinfection, which means a germ count reduction of at least 10^–3^ times, which corresponds to a reduction of 1:1000 = 1 germ in 1,000 germs is still viable after application of the measure. The aim of disinfection is thus the elimination of infectivity, i.e. the state of "asepsis" is aimed for^18^. Commercially available surface disinfectants generally meet these requirements in full. However, correct application is a prerequisite, i.e. the effective concentration in the local environment, exposure time and, if necessary, mechanical manipulation of the surfaces to be disinfected must be correctly mapped in accordance with the manufacturer's instructions under which the product was validated. This is where the "human bias" comes into play, i.e. the success of the application depends to a large extent on the way in which it is used. Different people use surface disinfectants in different ways, and there is also the general problem of personnel and time constraints, especially in the area of acute care (emergency services and general patient transport)^19^.

Furthermore, the use of wipes containing active ingredients, which has been propagated since the 2000s, poses a problem from the point of view of hygiene. The active ingredients, which are usually alcoholic or cationic surfactant-based, are only effective where application and wetting take place. Because of possible "overspray", the previously well-established spray disinfectants are no longer recommended^20^. This results in the problem of wetting gaps, since only the generally accessible surfaces are reached by application of the wipes. The wiping process does not reach certain, edges, beads, handles, blind holes, and folds. As a result, it is not possible to disinfect all functional areas in a reasonable time of about 30 min or less (since, for example, ambulances are needed or must be kept ready for missions), as required by the above requirement (reduction performance > 3 log levels).

The disinfection of ambulances is a fundamental requirement, typically conducted periodically or recurrently in alignment with the facility's hygiene plan, carried out from daily to weekly intervals. However, this process, as previously discussed, can be quite time-consuming when using wipes, especially given the need for comprehensive disinfection of all contact surfaces. For instance, in the case of an ambulance from the widely used Strobel Aalen system in Germany, this task demands approximately 2 h to complete. During this time, medical specialists are unavailable for immediate medical procedures, which are often urgently required. Instead, they carry out a basic medical procedure for two hours. In specific instances of direct contamination, such as the leakage of blood from a pressurized vein during the placement of a peripheral indwelling access, only limited disinfection of small surfaces is practical. The disinfection can be performed within minutes and is incomparable to the more extensive periodic disinfection process.

While the indicated disinfection of small surfaces as secondary or tertiary prevention, precisely indicated after the occurrence of contamination, can be effectively carried out immediately in a short time, there are great uncertainties with regard to periodic disinfection in the sense of primary prevention, i.e. the general reduction of infection risks, without directly knowing the cause (this is the decisive difference to the indicated disinfection of small surfaces, which must specifically cover a small manageable area, which is usually precisely known)^19^.

Therefore, from the point of view of systematic, technical hygiene, periodic disinfection, considering the time axis and the "man-power" performing it, is a non-validated quantity and therefore to be considered uncertain. In the absence of other methods, only periodic disinfection/basic disinfection has been possible to date, and this is time-consuming and never completely feasible due to the nature of the application (human bias/wetting gaps due to wipes). Therefore, this process cannot be validated and is unsafe^21^.

Up to this point, this discrepancy had to be accepted, considering that the patient's risk of mortality is typically higher when emergency treatment is provided than the risk of infection spread. Nonetheless, this interpretation, though reasonable, does not address the potential infection risk faced by the personnel who carry out essential medical procedures on the patient. Even alternative procedures of periodic disinfection do not close the "disinfection gap" between the applications of the just periodic disinfection procedure. One such procedure is hydrogen peroxide aerosol disinfection^21,22^. While a significantly greater wetting depth is attained through the primarily mechanical application of an aerosol compared to manual wipe disinfection, this process can only be conducted in the absence of staff and patients, e.g. on a periodic basis. Moreover, these measures are more time-consuming than manual wipe disinfection, as they require consideration in of both contact time and ventilation time. Consequently, the vehicle's downtime during this process is extended, often requiring 3 to 4 h for complete disinfection.

An increased risk of infection remains due to the fact that rapid, indicative disinfection, periodic wipe disinfection, and even periodic disinfection using apparatus, such as hydrogen peroxide aerosol, cannot fill the above-mentioned ''disinfection gap''. The plasma disinfection process within the PLASMOCAR device can bridge this ''disinfection gap''. It releases disinfection-active components through an electrophysical reaction involving atmospheric oxygen and water vapor in the air. Compared to filters, the air does not have to be conveyed through the device at a defined exchange rate. Rather, the disinfection-active components are emitted into the room air, which leads to "Microbe stimulation" in the air and the surfaces in contact with air. This breaks aerogenic infection chains and smear infections (primarily fecal–oral and hematogenic in this case) infection chains^16,23,24^.

The process can be applied continuously, ensuring ongoing disinfection of surfaces. Wetting gaps become irrelevant in this context, as the disinfection-active components diffusely disperse throughout the ambulance's room air, reaching all surfaces, including those that would be impractical to reach through manual wipe disinfection within a reasonable timeframe. Additionally, the "human factor" is eliminated, and the procedure can be standardized and validated procedure, analogous to disinfection of dishes or instruments using equipment. The objective of this study was to demonstrate potential applications and constraints of cold plasma for disinfection within emergency medical services. The PLASMOCAR from WK-Medtec GmbH was used for this purpose.

## Methods

Enterococcus faecium is used to test the disinfection efficacy of certain disinfection procedures on surfaces (EN 14476; EN 17272). This strain is non-pathogenic but displays resistance to inactivation to such an extent that its elimination includes the eradication of the relevant pathogenic bacteria, fungi, and enveloped viruses, or represents them cumulatively. This pathogen is cultured in a solution and then applied as a suspension to the surface in question using a sterile swab (referred to as "test contamination"). The contaminated area is bisected by applying a marker. After the test contamination has dried, the bacterial count on one half of the contaminated surface is assessed before disinfection. Following the completion of the disinfection process, the bacterial count on the other half of the surface is determined. Since surfaces are made of different materials and the materials have different properties, a sufficient selection of materials is made in the ambulance. Surfaces are composed of materials such as plastic, stainless steel, synthetic fiber textile fabric, synthetic leather, and baked enamel/epoxy resin paint on metal. Furthermore, the surfaces are selected in such a way that surfaces close to the PLASMOCAR unit, as well as surfaces further away, and internal surfaces within undercuts (e.g. the inside of drawers) are included in the test. The following explanations provide an overview of the practical definition of surfaces in the current investigation^25^.

Qualitative Overview Investigation of Surface Germ Count Density for Trend Description:

In practical surface monitoring (functional surfaces in ambulances, stations, commercial kitchens, supply air ducts of ventilation systems, and various other applications), the aerobic, mesophilic colony count is typically determined using the smear stamp method. For this purpose, nutrient agar is filled to the brim in special Petri dishes (RODAC plates = Rapid Organisms Detecting and Counting) in a way that the nutrient medium slightly extends beyond the upper edge due to capillary depression. These plates have a surface area of 25 cm2. By pressing the surface of the nutrient medium against the surfaces to be tested, the microorganisms present there are transferred onto the nutrient media and can be counted as colonies after incubation. A non-selective nutrient agar was used to determine the aerobic, mesophilic colony count. Here growth of other microorganisms besides the test germ Enterococcus faecium is to be expected. Specifically, endospore-forming aerobic bacteria (e.g., Gram-positive, endospore-forming aerobic bacteria such as Bacillus spp. or molds) can potentially outgrow the culture medium, thus complicating and interfering with the evaluation of the intended targets, i.e., the enterococci colonies. Nevertheless, even with these potential interferences, a trend can be identified when evaluating a sufficient number of samples, provided that the disinfection effect of the tested process is given. Unfortunately, achieving absolute quantification in this case remains impossible.

### Detailed, quantitative investigation of disinfection effects through surface dressing tests and subsequent dilution series

In addition to this investigation, a quantitative and reproducible microbiological investigation is carried out. To accomplish this, the previously defined and contaminated surface is swabbed using a sterile swab moistened with a 0.9% NaCl solution. This way the ''initial bacterial count'' is determined. Then the disinfection procedure is applied. After completion of the procedure, the second part of the surface is swabbed in the same way with a sterile moistened swab. The swabs are preserved in test tubes. In the laboratory, the test tubes containing the swabs are covered with 10 mL of sterile NaCl solution and homogenized using a Vortex(R) shaker. This action re-suspends the microorganisms collected on the swab. From each of these fractions, a dilution series is applied in powers of ten to 10^–3^. For each dilution step, 100µL is applied to a Slanetz-Bartley agar (enterococcus-selective culture medium) and homogeneously distributed using a Drigalski spatula. After incubation at 44 °C for 3 days, the colony count of the culture media is collected. The culture medium showing 10 to 100 colonies is used for counting. Considering the applied volume (here: 100µL of 10 mL) and the dilution level, the bacterial count of the test surface is calculated. From the determined microbial counts per unit area (microbial count density), the decadic logarithm is calculated, which converts the actual exponential decrement kinetics into a linear and proportional comparison. Therefore, the logarithmic value of the microbial count after disinfection can be subtracted from the logarithmic value of the microbial count before disinfection. In this way, the log reduction factor is obtained, which is the measure of the effect dynamic strength of the tested process (EN 14476, EN 17272).

## Results

**Figure 2:**
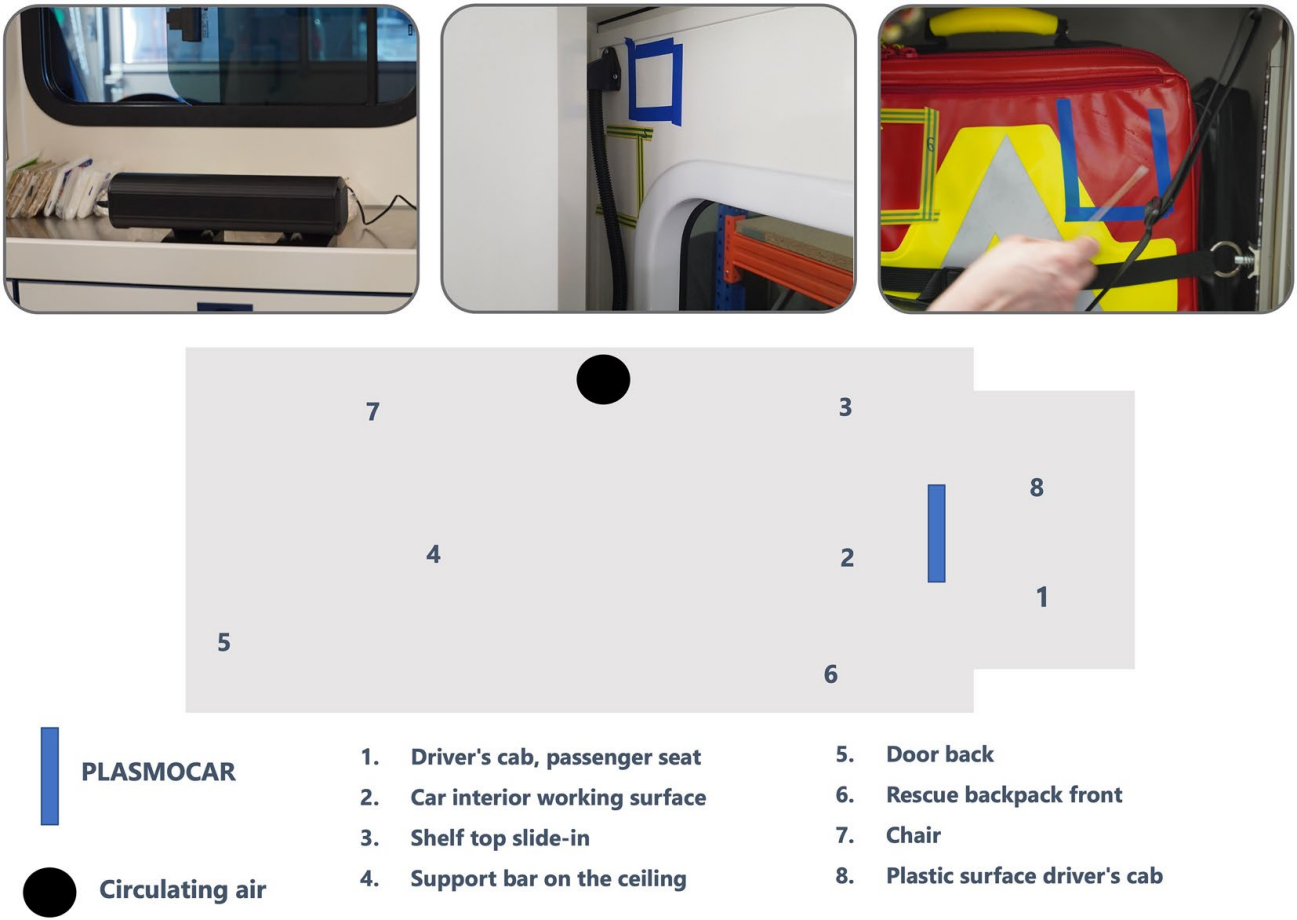
Test procedure and contaminated areas.

In a field test with 8 test surfaces in an ambulance of the German Red Cross Chemnitz on December 13, 2022, the PLASMOCAR device shows a significant Microbe stimulating or pathogen activating effect with regard to the test microorganism Enterococcus faecium (Fig. 2). After 30 min, a reduction of the initial microbial load by 3.73 log levels was achieved for Enterococcus faecium in the field test on the 8 test surfaces in the ambulance. This corresponds to a reduction in the bacterial count by a factor of approximately 10^–4^, meaning that up to 99.99% of the initial microorganisms have been eliminated (Table 1).

**Table 1:** Microbiology test results.

No.:	Volume of dilution step	Volume inoculated on plate	CFU/plate	CFU/dilution step	Dilution factor	CFU/examined surface	Log CFU/examined surface	Log reduction value (LRV)	Average Log LRV
Enterococcus faecium; ATCC No.6057; origin: DSMZ - Deutsche Sammlung für Mikroorganismen und Zellkulturen, Brunswick, Germany									
CFU/examined surface *initial* (before applying the examined plasma disinfection process). Examined surface was 8*8 cm for each test 1 until 8									
1	10.0 mL	0.1 mL	34	3400	10,000	34,000,000	7.53		
2	10.0 mL	0.1 mL	59	5900	10,000	59,000,000	7.77		
3	10.0 mL	0.1 mL	42	4200	10,000	42,000,000	7.62		
4	10.0 mL	0.1 mL	99	9900	10,000	99,000,000	8.00		
5	10.0 mL	0.1 mL	14	1400	10,000	14,000,000	7.15		
6	10.0 mL	0.1 mL	26	2600	10,000	26,000,000	7.41		
7	10.0 mL	0.1 mL	33	3300	10,000	33,000,000	7.52		
8	10.0 mL	0.1 mL	21	2100	10,000	21,000,000	7.32		
CFU/examined surface *final* (after applying the examined plasma disinfection process). Examined surface was 8*8 cm for each test 1 until 8									
1	10.0 mL	0.1 mL	39	3900	1	3900	3.59	3.94	
2	10.0 mL	0.1 mL	76	7600	1	7600	3.88	3.89	
3	10.0 mL	0.1 mL	98	9800	1	9800	3.99	3.63	
4	10.0 mL	0.1 mL	91	9100	1	9100	3.96	4.04	
5	10.0 mL	0.1 mL	43	4300	1	4300	3.63	3.51	
6	10.0 mL	0.1 mL	56	5600	1	5600	3.75	3.67	
7	10.0 mL	0.1 mL	81	8100	1	8100	3.91	3.61	
8	10.0 mL	0.1 mL	54	5400	1	5400	3.73	3.59	3.73

## Discussion

Disinfection describes a process which puts the objects or surfaces to be disinfected into a condition from which there is no longer any risk of infection^19^.

According to the professional societies for hygiene and microbiology in Germany and Europe, this is achieved when a 1,000-fold (10–3 log RF 3) to 100,000-fold (10–5 log RF 5) reduction in the number of germs is achieved.

Then the few microorganisms remaining on the surfaces no longer pose a risk of infection. The state of absence of a risk of infection is called "asepsis". Disinfection measures are therefore antiseptic measures that produce the state of asepsis^18^.

The results exemplarily show a homogeneous distribution in a scatter interval of—absolute – 0.5 log—levels. Therefore, the use of "only" 8 surfaces as a sample number can be justified. Simultaneously, the results provide no evidence of a location-specific dependency regarding the impact of the plasma reaction products. This can be explained by the saturation of the air with disinfection-active plasma reaction products and the recirculation function of the ventilation system. The field test has shown that the direct application and the continuous application of the device PLASMOCAR achieves a microbial count reduction on complex surfaces inside an ambulance by about 99.99%, showing that the device and the process used in it (atmospheric low-temperature plasma with the reaction partners oxygen and water vapor in the gas phase of the air) creates the state of asepsis on surfaces in the near field of the device, or on surfaces inside a room. The results obtained show that the process reproduces disinfection in the sense of asepsis described previously. Already after 30 min this condition is reached. The fact that the device is intended to operate permanently ensures that this state is constantly present. The test pathogen Enterococcus faecium is a proven tracer pathogen for testing disinfection procedures according to the standards described at the beginning. The sufficient inactivation of Enterococcus faecium shows that the tested procedure is effective against the relevant pathogenic bacteria and fungi (effect class A according to Robert Koch Institute). Furthermore, the tested procedure is also effective against enveloped, lipophilic viruses (e.g. HI virus, Hepatits C virus, SARS-CoV-2 virus, etc.), (effect class B as limited virucide against enveloped viruses). Therefore, taking into account the microbiological results obtained, it can be stated that the tested method/device (PLASMOCAR) leads to aseptic surfaces in the treated ambulance when operated for more than 30 min and during continuous operation. Accordingly, the working hypothesis stated at the beginning can be confirmed. For this reason, the procedure can be used as a "background" procedure to support and relieve the existing and established disinfection procedures.

The existing disinfection procedures according to the hygiene plan of the facility are still to be implemented with regard to cleaning, since the mechanical component of cleaning surfaces is also achieved here.

The use of PLASMOCAR enhances safety and significantly reduces the background risk of microorganism transmission through smear infections and aerosols. Thus, the PLASMOCAR device closes the "disinfection gap" described at the beginning. However, the device doesn't merely bridge a gap; instead, PLASMOCAR serves as the foundation for surface disinfection in ambulances. Mechanical cleaning still needs to be carried out, when required.

## Data Availability

All data generated or analyzed during this study are included in this published article.
