# Author Correction: Plasma disinfection procedures for surfaces in emergency service vehicles: a field trial at the German Red Cross

**DOI:** 10.1038/s41598-024-54622-8

**Published:** 2024-02-21

**Authors:** Tom Schaal, Ulrich Schmelz

**Affiliations:** 1 University of Applied Sciences Zwickau, Zwickau, Germany; 2 University of Fulda, Fulda, Germany

Correction to: *Scientific Reports* 10.1038/s41598-023-47759-5, published online 25 November 2023

The original version of this Article contained an error in Figure 2, where the black circle representing ‘Circulating air’ was inadvertently omitted. The original Figure 2 and the accompanying legend appear below.

**Figure 2:**
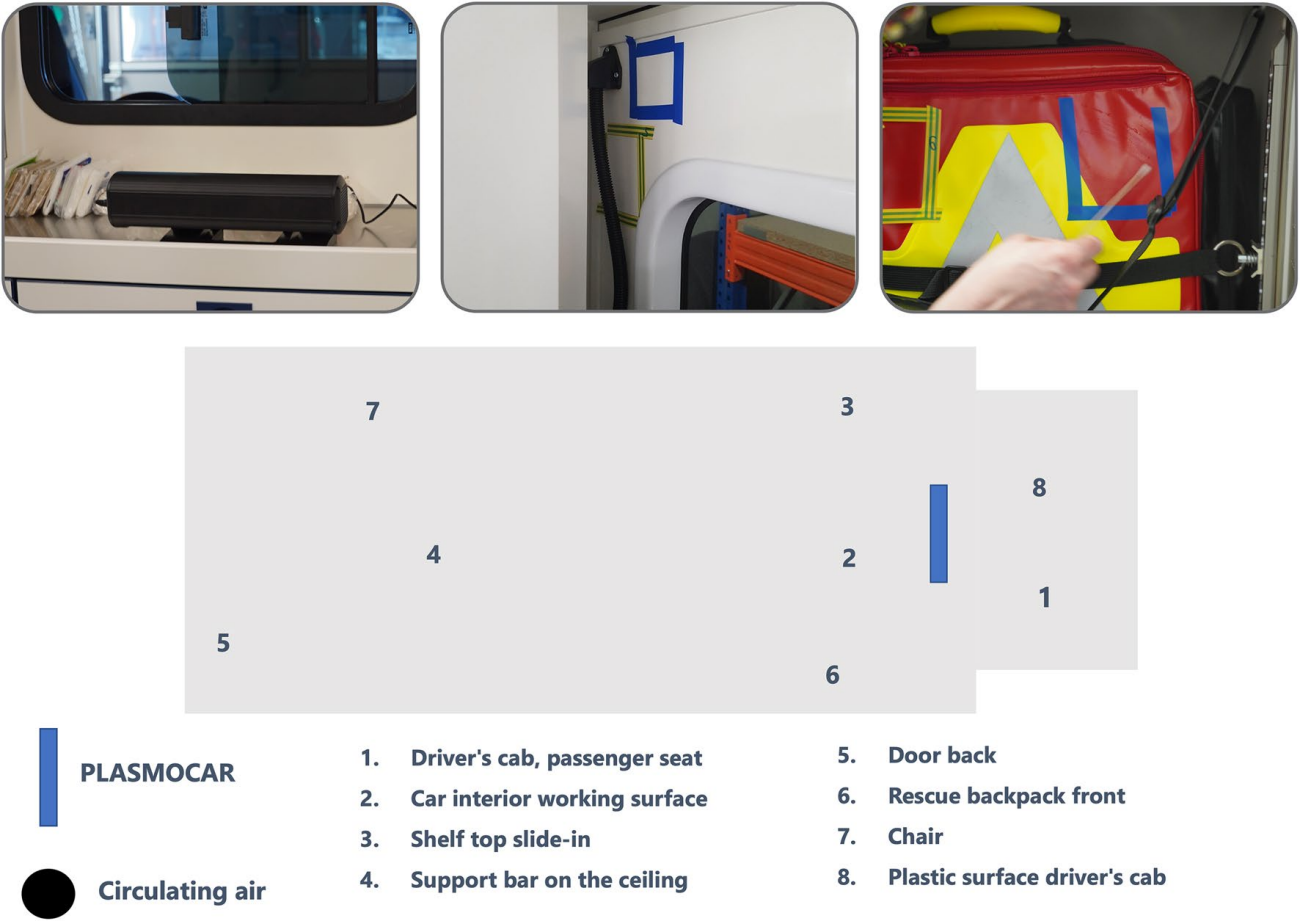
Test procedure and contaminated areas.

The original Article has been corrected.

